# The use of spectrograms improves the classification of wheezes and crackles in an educational setting

**DOI:** 10.1038/s41598-020-65354-w

**Published:** 2020-05-21

**Authors:** J. C. Aviles-Solis, I. Storvoll, Sophie Vanbelle, H. Melbye

**Affiliations:** 1 General Practice Research Unit, Department of Community Medicine, UiT The Arctic University of Norway, Tromsø, Norway; 2 Faculty of Health Sciences, UiT, The Arctic University of Norway, Tromsø, Norway; 3 Department of methodology and statistics, University of Maastricht, Maastricht, The Netherlands

**Keywords:** Physical examination, Respiratory signs and symptoms

## Abstract

Chest auscultation is a widely used method in the diagnosis of lung diseases. However, the interpretation of lung sounds is a subjective task and disagreements arise. New technological developments like the use of visSual representation of sounds through spectrograms could improve the agreement when classifying lung sounds, but this is not yet known. In this study, we tested if the use of spectrograms improves the agreement when classifying wheezes and crackles. To do this, we asked twenty-three medical students at UiT the Arctic University of Norway to classify 30 lung sounds recordings for the presence of wheezes and crackles. The sample contained 15 normal recordings and 15 with wheezes or crackles. The students classified the recordings in a random order twice. First sound only, then sound with spectrograms. We calculated kappa values for the agreement between each student and the expert classification with and without display of spectrograms and tested for significant improvement between these two coefficients. We also calculated Fleiss kappa for the 23 observers with and without the spectrogram. In an individual analysis comparing each student to an expert annotated reference standard we found that 13 out of 23 students had a positive change in kappa when classifying wheezes with the help of spectrograms. When classifying crackles 16 out of 23 showed improvement when spectrograms were used. In a group analysis we observed that Fleiss kappa values were k = 0.51 and k = 0.56 (p = 0.63) for classifying wheezes without and with spectrograms. For crackles, these values were k = 0.22 and k = 0.40 (p = <0.01) in the same order. Thus, we conclude that the use of spectrograms had a positive impact on the inter-rater agreement and the agreement with experts. We observed a higher improvement in the classification of crackles compared to wheezes.

## Introduction

Chest auscultation is a widely used method in the diagnosis and follow up of several diseases. Health professionals use it to guide clinical decisions and treatment strategies^1^. However, the identification and interpretation of the sounds remains a subjective task. Since stethoscopes are designed for individual listening it is not possible to share the sounds with other colleagues for discussion. Often, disagreement between health professionals arises when classifying lung sounds and the reliability in the classification of wheezes and crackles has been found to be moderate at best^2–6^. This variation is caused not only due to health professionals failing to identify lung sounds, but also due to different labeling of the sounds^7,8^. The inability of the traditional stethoscope for collective hearing also creates situations where patients with an uncommon heart or lung sound will be repetitively auscultated by numerous students who are only interested in “the sound”. This might have an impact on the patients making them feel as objects^9^. These limitations of traditional auscultation make the training of new health professionals a challenging task. It has been suggested that difficulties in teaching and learning auscultation also contribute to the demise of this technique^10^.

**Figure 1:**
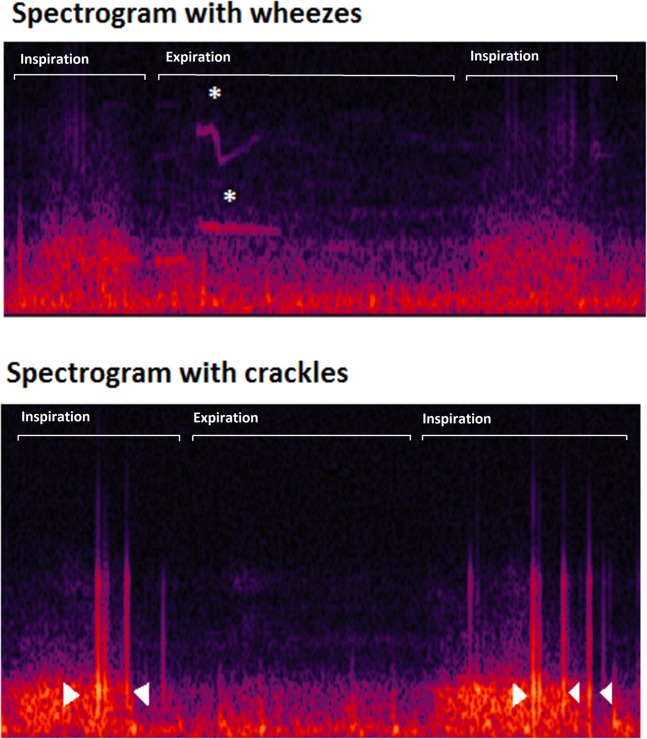
Examples of spectrograms of lung sound recordings showing the presence of wheezes (stars) and crackles (arrowheads).

However, new electronic stethoscopes can capture and store sounds in a digital form. This opens many possibilities to analyze and share lung sounds. One of these possibilities is the generation of spectrograms from digital lung sounds recordings. A spectrogram is a visual representation of the spectrum of frequencies of a signal as it varies with time. In spectrograms, adventitious lung sounds, such as wheezes and crackles, show recognizable patterns, (Fig. 1) which may be of help in the identification of these sounds^11^.

It would be natural to think that the use of a visual support could help to improve the classification of lung sounds since there are two sensory inputs instead of one. The use of spectrograms could improve the teaching of lung sounds by giving an aid to the listening abilities in training. Andrés *et al.*^12^ found that the spectrograms do have a positive impact on how medical students assign a diagnosis with the help of lung sounds. However, the design of this study did not isolate the effect of spectrograms and therefore this affirmation is inconclusive. Therefore, we do not know whether the use of spectrograms would help medical students to better classify lung sounds. If spectrograms have a positive effect in the classification of lung sounds it would then help make auscultation training more simple and effective.

The aim of this study is to explore how the use of spectrograms affects the agreement between medical students and a panel of experts and the agreement within a group of students in classifying lung sounds.

## Methods

### Data for classification

The sound recordings employed in this study were also used in the study of Aviles-Solis *et al.*^13^. The description of the sound recording method is therefore the same as in the mentioned study. We recorded lung sounds from 20 adults. We took contact with a rehabilitation program in northern Norway for patients with heart and lung-related diseases (lung cancer, chronic obstructive pulmonary disease, heart failure). We got permission to hold a presentation about lung sounds and at the end of the presentation we invited the patients to be part of our research project. Fourteen patients attending the rehabilitation program agreed to participate and we recorded the lung sounds that same evening. The patients were 67.43 years old on average (44–84) and nine were female. To hold a balanced sample (concerning the prevalence of wheezes, crackles and normal lung sounds), we obtained the rest of our recordings from six self-reported healthy employees at our university aged 51.83 years old on average (46–67) and five were female. We registered the following information about the subjects: age, gender and self-reported history of heart or lung disease.

To record the lung sounds, we used a microphone MKE 2-EW with a wireless system EW 112-P G3-G (Sennheiser electronic, Wedemark, Germany) placed in the tube of a Littmann Master Classic II stethoscope (3 M, Maplewood, MN, USA) at a distance of 10 cm from the headpiece. The microphone was connected to a digital sound Handy recorder H4n (Zoom, Tokyo, Japan).

**Figure 2:**
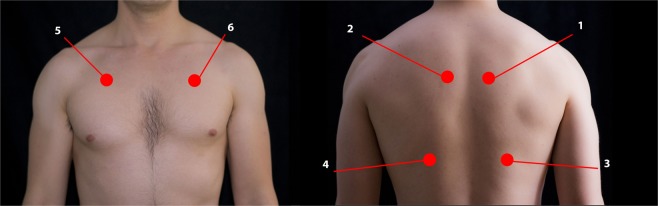
Illustration showing the different places where lung sounds were recorded. (1_2) Between the spine and the medial border of the scapula at the level of T4–T5; (3_4) at the middle point between the spine and the mid-axillary line at the level of T9–T10; (5_6) at the intersection of the mid-clavicular line and second intercostal space.

We placed the membrane of the stethoscope against the naked thorax of the subjects. We asked the subjects to breathe deeply while keeping their mouth open. We started the recording with an inspiration and continued for approximately 20 seconds trying to capture three full respiratory cycles with good quality sound. We performed this same procedure at six different locations (Fig. 2). The researcher recording sounds used a headphone as an audio monitor to evaluate the quality. When too much noise or cough was heard during the recording, a second attempt was performed.

We obtained 120 audio files in ‘.wav’ format and recorded at a sample rate of 44 100 Hz and 16 bit depth in a single monophonic channel. We did not perform post-processing of the sound files or implement filters.

We chose 30 recordings for this study from 18 different subjects. This selection contained 15 normal and 15 abnormal sounds, from which nine were classified as containing crackles and six containing wheezes. The reason to choose 30 recordings was more in relationship with the amount of time we thought the students would invest in the study rather than a statistical calculation. Considering the logistics of the study, we estimated that classifying 30 sound recordings twice would take a total of two hours. We thought it would be hard to get volunteers to be there more than two hours.

A panel of four experts in the field of lung sound research classified the recordings according to the presence of wheezes and crackles. We decided that adventitious lung sounds (wheezes or crackles) were present if three out of four experts agreed on it.

### Observers

We invited medical students from third to fifth year at UiT, The Arctic University of Norway to participate in the study. The students interested in participating sent an e-mail requesting to be included in the study. We were very clear to the students that they were free to withdraw from the study whenever they wanted without any consequence for them. The students had received the standard curricular training in physical examination provided in the medicine school. The training in lung auscultation included a lecture on lung sounds which included the demonstration of some spectrograms. Beyond this, the students had no previous experience with the use of spectrograms.

### Presentation and classification of the lung sounds

First, we presented to the whole group with one recording of normal lung sounds, one recording of wheezes and one of crackles. The recordings were accompanied by its respective spectrogram and we explained the students how to identify wheezes or crackles in the spectrograms. We explained that all recordings started with an inspiration. The explanation took around four minutes. The students were not given the opportunity to practice. After the explanation we played the 30 recordings containing only sound in a random order. We then played the same recordings in a random order but this time accompanied by its respective spectrograms displayed in the classroom screen. The recordings were played in a different random order in both sessions. There was a pause of 20 minutes in between the two sessions. We presented no additional information beyond the sound and the spectrograms. The observers were not aware that the same sounds were played in both sections.

In both sessions, each recording was played two times and the students had up to 30 seconds to classify it before moving to the next recording. The observers used their personal computers and an online classification scheme (Questback AS, Norway). In this scheme, the observers had to specify if the recording contained only normal respiratory sounds. If this was not the case, the observers had to further specify if the recording contained wheezes, crackles or other sounds and if they appeared during inspiration or expiration. It was also possible to mark the recording as containing too much noise to be classified. At the end of the classifications session we obtained a report in an excel document. (Microsoft, Redmond, WA, USA).

### Statistical analysis

We calculated four Cohen kappa coefficients (k=) for each participant. Two of them for the agreement of wheezes between each of the observers and the annotation of the experts (Without and with spectrograms). The same for crackles. We then compared these two kappa values for each observer (Wheezes without spectrograms vs. wheezes with spectrograms and crackles without spectrograms vs. crackles with spectrograms). We calculated the difference between these two coefficients and summarized it in a graph. We also calculated p values to explore for statistically significant differences using an adaption of Hotelling’s T^2^ test described by Vanbelle, S^14^. Since we were testing the same hypothesis many times we used Holm’s correction procedure to adjust the obtained p values for multiple hypothesis testing.

In a similar fashion we calculated four Fleiss kappa coefficients (k=) for all the observers as a group annotating wheezes and crackles without and with spectrograms. We calculated the difference between them, calculated 95% confidence intervals and tested for statistical significance with the aforementioned statistical method. In the agreement analysis (both Cohen and Fleiss), the recordings were clustered by the individual they were recorded from.

We tested for significant differences in sensitivity and specificity of each observer (with and without spectrograms) using paired Wilcoxon signed rank test with continuity correction. We used the experts’ classification as the gold standard.

We used R version 3.2.1 and the package “magree” to perform all the calculations. Significance level was set at p < 0.05.

The results of this study are reported according to the Guidelines for Reporting Reliability and Agreement Studies (GRRAS)^15^.

### Ethical approval and informed consent

The project was presented for the Regional Committee for Medical and Health Research Ethics of Norway (REC South East). The committee waived to evaluate the project since it was considered to be outside the remit of the Act on Medical and Health Research and a written consent was not deemed necessary. No personal information was registered that could link the collected data to the individual subjects. All the participants provided verbal consent. All the methods in the study were carried in accordance with current ethical guidelines.

## Results

### Observers

We included 23 observers in the study. At the beginning, 30 students accepted to participate in the study. From them, two withdrew before the start of the study, two did not show up and three did not complete the classification session due to lack of time. Eight participants were third year students, fourteen participants were from the fourth year and one was from the fifth year. There were 19 women and four men.

### Agreement

The students observed a mean prevalence of wheezes of 9.7 (6–15) without spectrograms and 8.3 (5–12) with spectrograms. In the case of crackles the students observed a mean prevalence of 11.5 (4–22) and 10.9 (5–18) in the same order. The mean proportion of agreement (%) and Cohen kappa (k) with the experts for all 23 participants classifying wheezes without spectrograms was 82% and k = 0.56. We observed 88% and k = 0.68 with the use of spectrograms. In the case of crackles we observed a proportion of agreement of 72% and k = 0.38 without spectrograms and 80% and k = 0.56. with the use of spectrograms.

**Figure 3:**
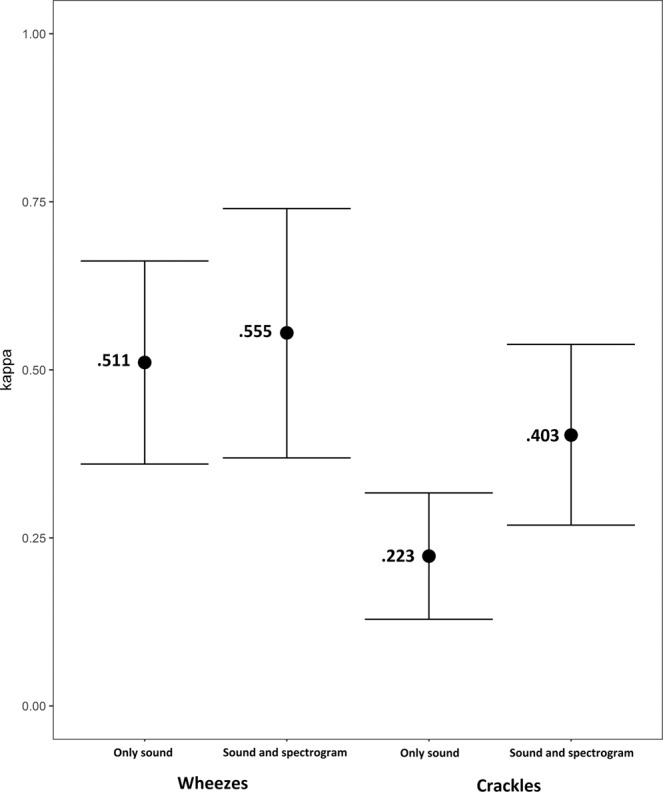
Fleiss kappa for the group of 23 participants when classifying wheezes and crackles with only sound and sound plus spectrogram.

Fleiss kappa values for the multirater agreement were k = 0.51 and k = 0.56 (p = 0.63) for wheezes without and with spectrogram, respectively. For crackles, we observed k = 0.22 and k = 0.40 (p = <0.01) in the same order. (Fig. 3).

**Figure 4:**
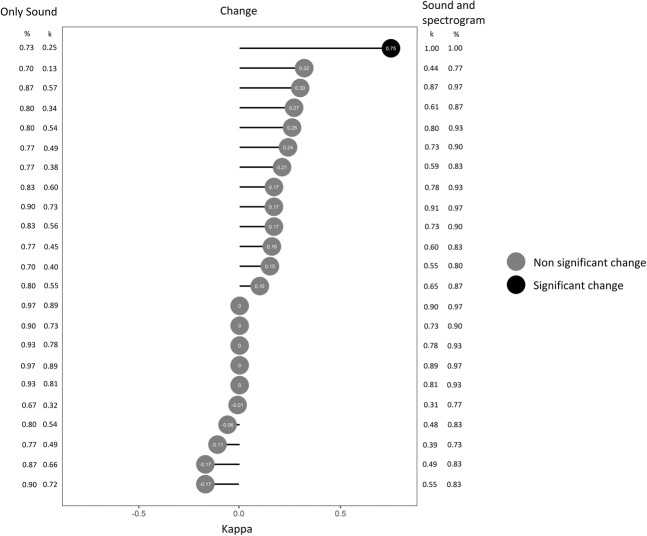
Cohen’s kappa of each participant when classifying wheezes with only sound (left) and with sound and spectrograms (right) compared to the reference standard. The change in kappa between the two classifications and its statistical significance is illustrated at the center. Proportion of agreement (%) is presented on the lateral columns.

**Figure 5:**
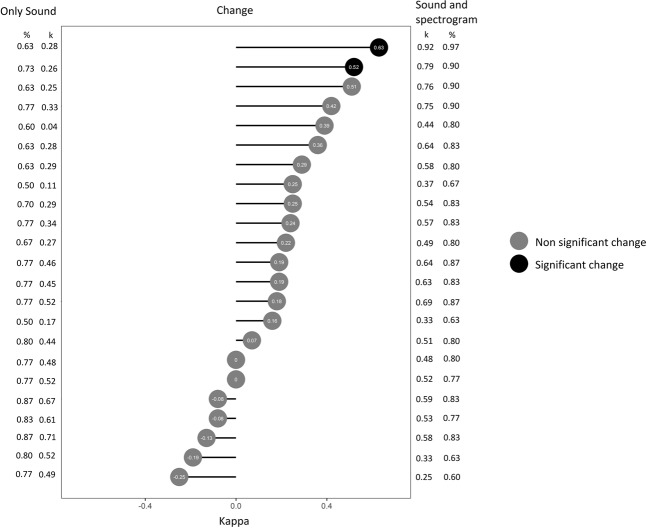
Cohen’s kappa of each participant when classifying crackles with only sound (left) and with sound and spectrograms (right) compared to the reference standard. The change in kappa between the two classifications and its statistical significance is illustrated at the center. Proportion of agreement (%) is presented on the lateral columns.

Compared to the expert panel’s classification, 13/23 students had a positive change in kappa when classifying wheezes (one with p < 0.05), and 16/23 (two with p < 0.05) when classifying crackles (Figs. 4 and 5). All the statistically significant changes were in the direction of improved kappa values (0.52–0.75).

When looking at the classification of normal vs abnormal sounds (wheezes or crackles) we observed a mean prevalence of abnormal sounds of 18.7 and 18 without and with spectrogram (experts 15). The mean absolute agreement was 72% with a mean kappa of k = 0.44 without spectrograms and 80% and k = 0.60 with spectrograms. Only one participant had a significant improvement in this analysis.

**Figure 6:**
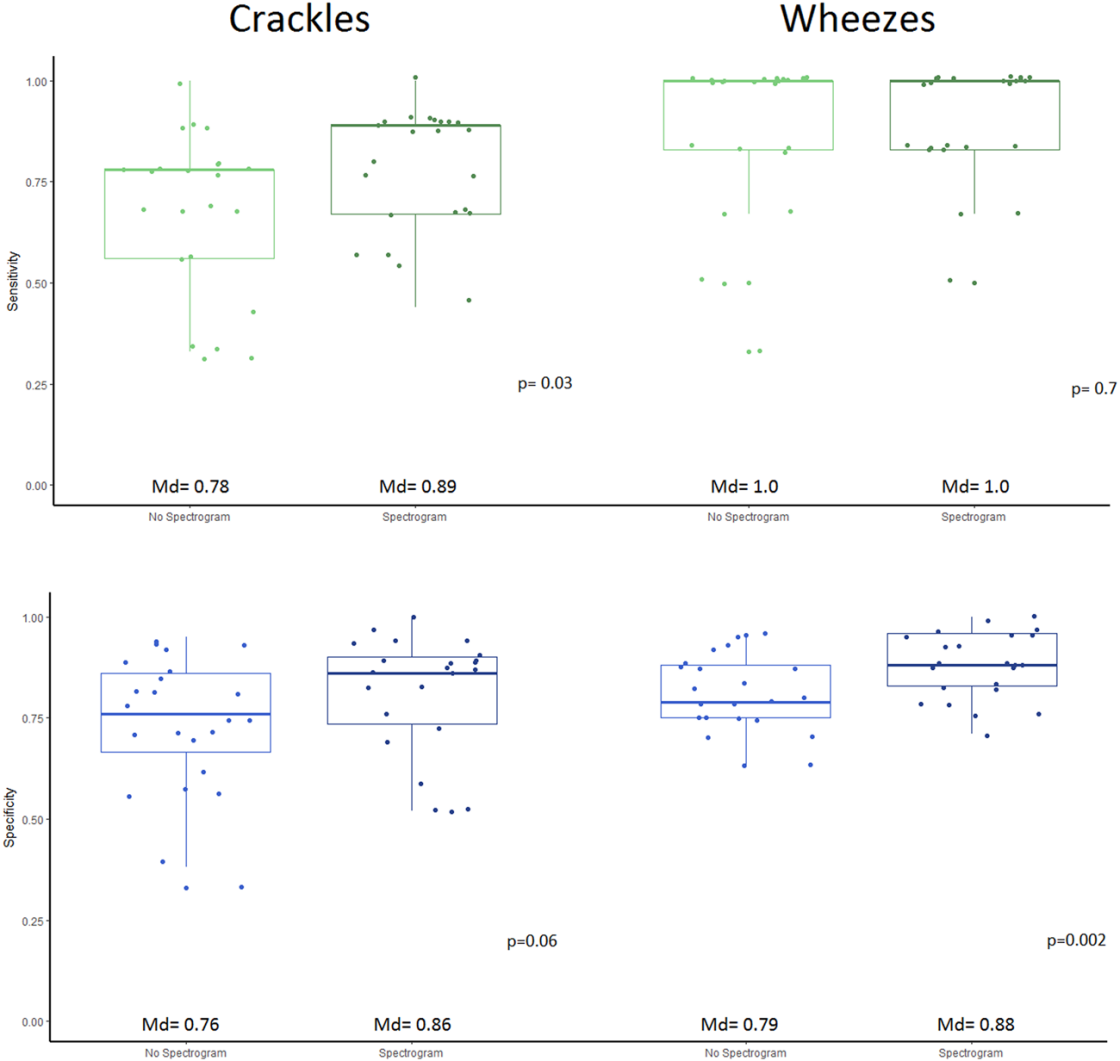
Box and whiskers diagrams showing the change in sensitivity and specificity of the students classifying wheezes and crackles with and without spectrograms. The answers of the experts were considered as the reference standard. P values shown were obtained from the test of difference between means using paired Wilcoxon signed rank test with continuity correction. Md= Median.

The median sensitivity for wheezes (100%) did not present a significant change but the specificity was higher in the classification with the use of spectrograms (79% vs 88%, p = 0.002). In the case of crackles, there was a significant increase in sensitivity (78% to 89%, p = 0.03) when using spectrograms but without significant change in specificity (76% to 86%, p = 0.06) (Fig. 6).

## Discussion

We found improved agreement with the experts in the classification of lung sounds with the use of spectrograms. However, most of the improvements were not statistically significant. We did observe a significant improvement in the within-group agreement (Fleiss kappa) when classifying for crackles when the sounds were presented with spectrograms.

Students from 4th 5th and 6th year had received a lecture on lung sounds and were showed spectrograms as part of their training. 3rd year students might have been in disadvantage for identifying adventitious lung sounds with spectrograms. That was the reason why we showed examples of lung sound recording with its respective spectrogram (one with wheezes and one with crackles) to all the participants in the study. By doing this, we think that the chance of bias was reduced. It could also be argued that the explanation at the beginning of the study could have had a positive bias making the students achieve better performance in classifying lung sounds. We do not believe this is a significant source of bias since the experiment was designed to measure the ability for pattern and sound recognition. In order to recognize a pattern/sound we need to explain the participants which pattern and or sound is expected to be recognized. Since the demonstration also included both pattern and sound, any eventual influence of this demonstration would have equally impacted both classification modes (with and without spectrograms) thus not affecting the comparison between the two different modes of classification.

The levels of individual and group agreement observed in this study corresponds with that reported in previous studies^13^. The agreement for wheezes was higher compared to crackles. This is in accordance with what is described in the literature^3^. It is interesting that the impact of spectrograms was different for wheezes and crackles. It might be that wheezes are easier to recognize without spectrograms due to its relatively long duration and its musical quality, which make them more familiar to the human ear. On the contrary, crackles are short explosive sounds that could easily be missed by ear appreciation or perceived as noise^16^. For this reason, having a visual aid could be an advantage to identify them.

Andrés *et al.*^12^ found that the spectrograms do have a positive impact on how medical students assign a diagnosis to a patient with the help of lung sounds. Even though the observer populations are similar, the results are not comparable since the outcome in their test was a diagnosis and not the classification of the sounds. Since the participants in Andrés’ study got clinical information together with the sound this could have influenced how they classified lung sounds. Nguyen et al. observed that the addition of clinical information has an effect on the classification of lung sounds and this effect is experience-dependent. In Nguyen’s study, the group classifying lung sounds without clinical information achieved similar scores classifying lung sounds regardless of clinical experience. When clinical information was provided, then the more experienced raters achieved higher scores^17^.

Our exploratory study suggests that the use of spectrograms might be helpful to improve the teaching of auscultation, mostly in the case of crackles.

### Strengths and limitations

We think the methodology employed allowed us to analyze the isolated effect of the spectrograms in the classifications.

The students had free seating. Even though absolute silence was required during the classification, we cannot fully rule out the possibility that the students could have influenced each other. This could have influenced the estimation of agreement in the group (multi-rater) agreement in a positive direction.

We have tested the same hypothesis 23 times. This increases the chance of making a type I error. We have taken this situation into account by correcting the p-values with Holm’s procedure.

Some limitations in our study concern the sample size of sounds to classify. Due to the exploratory nature of this study, we chose a number of sounds to be classified which could allow us to perform the whole procedure in a time window of two hours. We estimated that it would be hard to recruit participants or that the rate of dropout would be high if the study took longer than two hours. It is possible that we could have had observed more significant changes if the sound sample would have been larger. Future studies looking at the effect of spectrograms in agreement should take this into account in its design.

## Conclusion

The use of spectrograms had a positive impact on the inter-rater agreement and the agreement compared to experts. We observed a higher improvement in the classification of crackles compared to wheezes.

## Data Availability

The datasets used and/or analyzed during the current study are available from the corresponding author on reasonable request.
